# Development of Polymer Blend Electrolyte Membranes Based on Chitosan: Dextran with High Ion Transport Properties for EDLC Application

**DOI:** 10.3390/ijms20133369

**Published:** 2019-07-09

**Authors:** Shujahadeen B. Aziz, Muhamad H. Hamsan, Mohd F. Z. Kadir, Wrya O. Karim, Ranjdar M. Abdullah

**Affiliations:** 1 Advanced Polymeric Materials Research Lab., Department of Physics, College of Science, University of Sulaimani, Qlyasan Street, Sulaimani 46001, Kurdistan Regional Government, Iraq; 2 Komar Research Center (KRC), Komar University of Science and Technology, Sulaimani 46001, Kurdistan Regional Government, Iraq; 3 Centre for Foundation Studies in Science, University of Malaya, Kuala Lumpur 50603, Malaysia; 4 Department of Chemistry, College of Science, University of Sulaimani, Qlyasan Street, Sulaimani 46001, Kurdistan Regional Government, Iraq

**Keywords:** biopolymer electrolyte membranes, XRD analysis, FTIR study, Morphology, Impedance study, EDLC fabrication

## Abstract

Solid polymer blend electrolyte membranes (SPBEM) composed of chitosan and dextran with the incorporation of various amounts of lithium perchlorate (LiClO_4_) were synthesized. The complexation of the polymer blend electrolytes with the salt was examined using FTIR spectroscopy and X-ray diffraction (XRD). The morphology of the SPBEs was also investigated using field emission scanning electron microscopy (FESEM). The ion transport behavior of the membrane films was measured using impedance spectroscopy. The membrane with highest LiClO_4_ content was found to exhibit the highest conductivity of 5.16 × 10^−3^ S/cm. Ionic (*t_i_*) and electronic (*t_e_*) transference numbers for the highest conducting electrolyte were found to be 0.98 and 0.02, respectively. Electrochemical stability was estimated from linear sweep voltammetry and found to be up to ~2.3V for the Li^+^ ion conducting electrolyte. The only existence of electrical double charging at the surface of electrodes was evidenced from the absence of peaks in cyclic voltammetry (CV) plot. The discharge slope was observed to be almost linear, confirming the capacitive behavior of the EDLC. The performance of synthesized EDLC was studied using CV and charge–discharge techniques. The highest specific capacitance was achieved to be 8.7 F·g^−1^ at 20th cycle. The efficiency (*η*) was observed to be at 92.8% and remained constant at 92.0% up to 100 cycles. The EDLC was considered to have a reasonable electrode-electrolyte contact, in which *η* exceeds 90.0%. It was determined that equivalent series resistance (*R_esr_*) is quite low and varies from 150 to 180 Ω over the 100 cycles. Energy density (*E_d_*) was found to be 1.21 Wh·kg^−1^ at the 1st cycle and then remained stable at 0.86 Wh·kg^−1^ up to 100 cycles. The interesting observation is that the value of *P_d_* increases back to 685 W·kg^−1^ up to 80 cycles.

## 1. Introduction

Electrochemical capacitors are classified into three types: Pseudocapacitor, electrical double-layer capacitor (EDLC), and hybrid capacitor [1]. In pseudocapacitor, Faradaic process is involved as the energy storage mechanism with metal oxide and conducting polymer electrodes [2]. However, in EDLC, which is usually composed of two identical carbon-based electrodes, non-Faradaic process is envolved during the charge storage process, where the accumulation of charge at the double-layer occurs on the surface of carbon-based electrodes [3]. In the third type, i.e., in the hybrid capacitor, the energy storage mechanism is based on the combination of EDLC and pseudocapacitor, i.e., both Faradaic and non-Faradaic processes contribute. Among these capacitors, EDLC has shown to obtain through a straightforward fabrication process. Moreover, EDLC possesses valuable properties, such as long life cycle, high power density and light in weight [4]. In making such capacitor, activated carbon has been reported to be compatible with polymer electrolyte [5,6,7]. Several features of activated carbon, for instance, high surface area, high electrical conductivity, cost-effectiveness, and excellent chemical stability, make it preferable for EDLC applications [8].

Biopolymers are preferable over non-degradable synthetic polymers as host polymers in polymer electrolyte system because of their renewability, biocompatibility, and biodegradability [9,10]. They are usually extracted from natural resources, e.g., cellulose from plants [11], starches from potato, maize and cassava [12], chitosan from crustacean animals [13], carrageen from seaweed [14], and dextran from bacteria [15]. The last one is obtained by growing cultures of bacteria called *Leuconostocmesenteroides* in a medium filled with sucrose. Dextransucrase as an enzyme is excreted in the medium where excess sucrose had converted to dextran [16]. Dextran with 1,6-α-d-glucopyranosidic linkages is a biodegradable and a non-toxic polymer in the polymer backbone [17]. Regarding the backbone of dextran, it is clear to observe two main functional groups, such as hydroxyl and glycosidic bond, which possess electron lone pairs that contribute in the ionic conduction [18]. Herein, it is interesting that chitosan as one of the derivatives of amino polysaccharides called chitin produced from exoskeleton of crustaceans or insects. Both amine and hydroxyl functional groups enable chitosan to serve as one of ionic conductors [19].

Blending two or more polymers has motivated researchers to improve the characteristics of individual polymers. Hamsan et al. [20] have showed that potato starch-methylcellulose blend film is more amorphous in structure than both pure methylcellulose film and potato starch film. It is well-known that the amorphous region in polymer electrolyte is mainly responsible for ionic conduction [21]. As concluded in the report published by Tamilselvi and Hema [22], mechanical properties or structure stability of a material can be manipulated by polymer blending method. Blended polymer composites provide more sites for ionic complexation process to occur, which makes ionic conduction to be efficient than single polymer [23]. Based on our previous work [24], in 60 wt.% chitosan and 40 wt.% dextran blend system, the amorphous structure was shown to be dominant. Therefore, in this work, a similar percentage of chitosan and dextran has been blended along with incorporation of lithium perchlorate (LiClO_4_). In addition to that, thehighest relatively conducting electrolyte has also been used as electrode separators in the EDLC.

## 2. Results and Discussion

### 2.1. Structural (XRD and FTIR) Analysis

**Figure 1:**
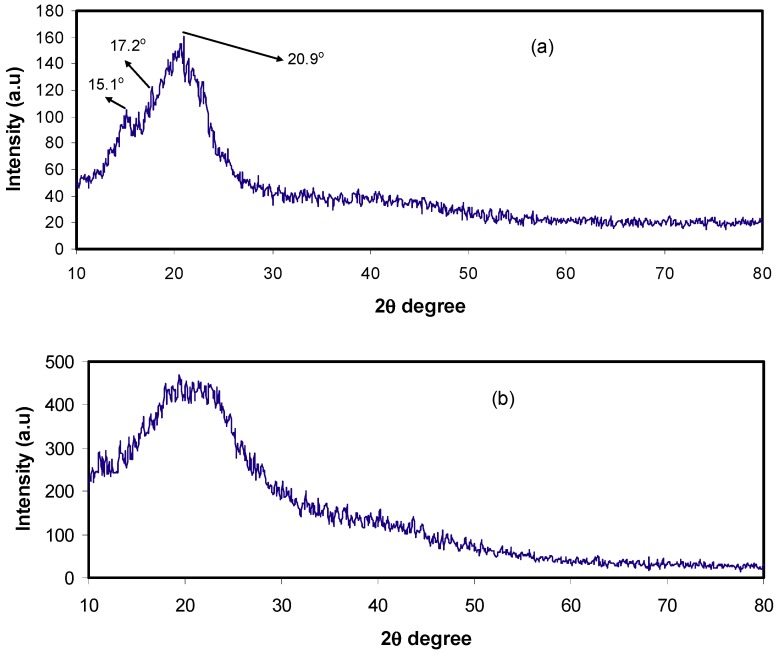
The XRD pattern for (**a**) pure CS and (**b**) CS:Dextran blend film.

**Figure 2:**
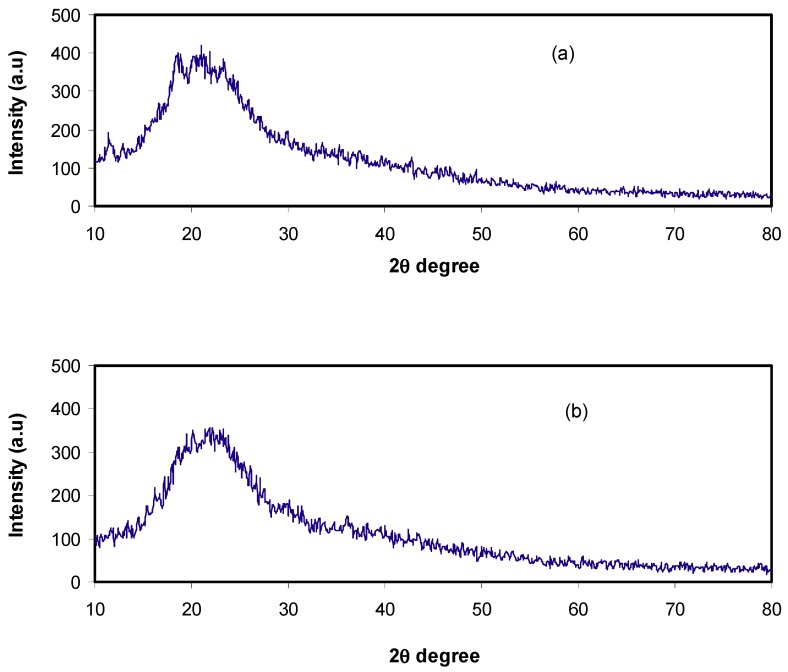
The XRD pattern for (**a**) CSDPB2 and (**b**) CSDPB4 blend electrolytes.

XRD analysis was carried out on pure CS, CS:Dextran and CS:Dextran: LiClO_4_ systems at the ambient temperature. In our previous work, it was shown that pure CS exhibits several crystalline peaks at 2θ = 15.1, 17.7, and 20.9° [25,26], as shown in Figure 1a, whereas Dextran exhibited two hollows at 2θ = 18 and 23° [27]. In the present work, the XRD pattern of CS:Dextran showed two hollows and no crystalline peaks can be observed, as can be seen in Figure 1b. These broad hollows indicate that a fully amorphous structure has been formed [28]. Earlier reports documented that a broad diffraction peaks corresponds to amorphous nature of the polymer electrolyte [29,30]. In the current study, it has been found that the intensity of the hump of CS:Dextrandecreases in the intensity with the addition of LiClO_4_ salt and broad nature also increases, as shown in Figure 2a,b. It is clear that at 20 wt.% of the salt some new peaks appeared, which are due to polymer slat complexes rather than pure salt. Interestingly, at 40 wt.% of the added salt, these new peaks disappeared and the intensity of the hump decreased. The results obtained here confirm the amorphous nature of the polymer electrolytes. The amorphous nature provides greater ionic diffusivity and high ionic conductivity. No peaks corresponding to pure LiClO_4_ appeared in the CS:Dextran blend polymer electrolyte, which indicates the complete dissociation of the dopant salt in the polymer blend matrix.

**Figure 3:**
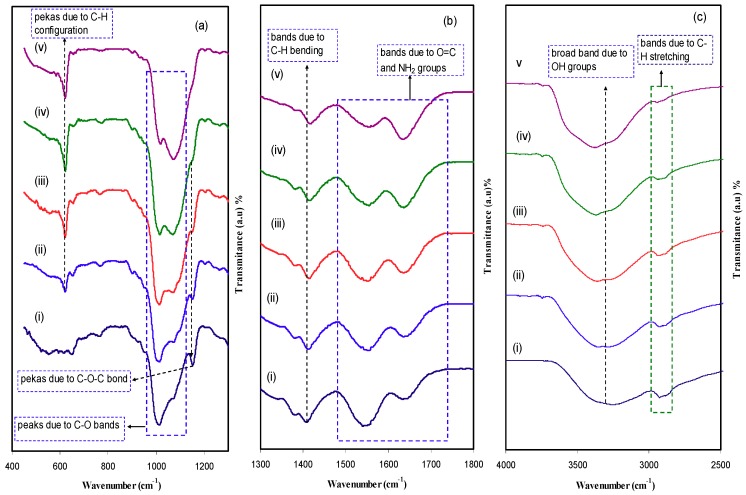
FTIR spectra of (i) CS:Dextran (pure film), (ii) CSDPB1, (iii) CSDPB2, (iv) CSDPB3, and (v) CSDPB4 in the region (**a**) 700 cm^−1^ to 1300 cm^−1^, (**b**) 1400 cm^−1^ to 1800 cm^−1^, and (**c**) 3000 cm^−1^ to 3800 cm^−1^.

Figure 3a–c shows the FTIR spectra of the pure CS:Dextran and blend electrolyte films at three separate regions. Fourier transform infrared (FTIR) spectroscopy has been widely used by many researchers in dealing with the formation of polymer blends. FTIR spectroscopy provides insight into intermolecular interaction through analysis of FTIR spectra on the basis of stretching or bending vibrations of particular bonds. The main characteristic bands in the spectra of dextran were found at 1146 and 1021 cm^−1^. The band peak at 1086 cm^–1^ is assigned to both valent vibrations of C–O–C bond and glycosidic bridge. The presence of a peak at 1021 cm^–1^ belongs to the high chain flexibility that present in dextran around the glycosidic bonds [31]. For CS:Dextran, the FT-IR spectrum exhibited the presence of OH groups that could be confirmed by the appearance of broad band with a maximum at 3351cm^−1^ [32,33]. In a comparison, the band peaked at 2906 cm^−1^ can be attributed to C–H stretching in dextran [32,33], since such band was not observed in the FTIR spectra of chitosan [34]. The peak at 1000 cm^−1^ characterizes the significant chain flexibility exist in dextran around the glycosidic bond [32]. The C–H bending usually appeared at 1450 cm^−1^, whereas the broad band starts at 1158 cm^−1^, indicating asymmetrical –C–O–C– stretching of the ring [33]. The sharp peak at 1009 cm^−1^ and small one at 1067 cm^−1^ is ascribed to the existence of C–O bands [34]. With increasing LiClO4 salt, these peaks are becomes distinguishable, as observed in our previous work for chitosan-based electrolyte. For C-H configuration in dextran, a characteristic peak appeared at 615 cm^−1^ [35]. For both stretching vibrations of the C–O–C bond and glycosides bridge a peak centered at 1155 cm^−1^. Two peaks centered at 1651 cm^−1^ and 1554 cm^−1^ corresponding to carboxamide (O=C–NHR) and amine (NH2) bands, respectively [34]. It is interesting to observe that a shift occurred in the carboxamide (O=C–NHR) and amine (NH_2_) bands which strongly confirms complexation between chitosan: Dxetran and the dopant salt. In fact, this attachment of cation salt to nitrogen and oxygen atoms can reduce the vibration intensity of the N–H or O=C–NHR bonds owing to the higher molecular weight after cation binding and eventually resulted in shifting and lowering in intensity [36]. More interesting observation is the incorporation of LiClO_4_ salt into CS:Dextran resulting in a great change in the intensity of the bands. This change in intensity of these bands is strongly related to the alterations in the macromolecular order. These bands in the spectra of the complexes may result from more and less ordered structures [37].

### 2.2. Morphological Study

**Figure 4:**
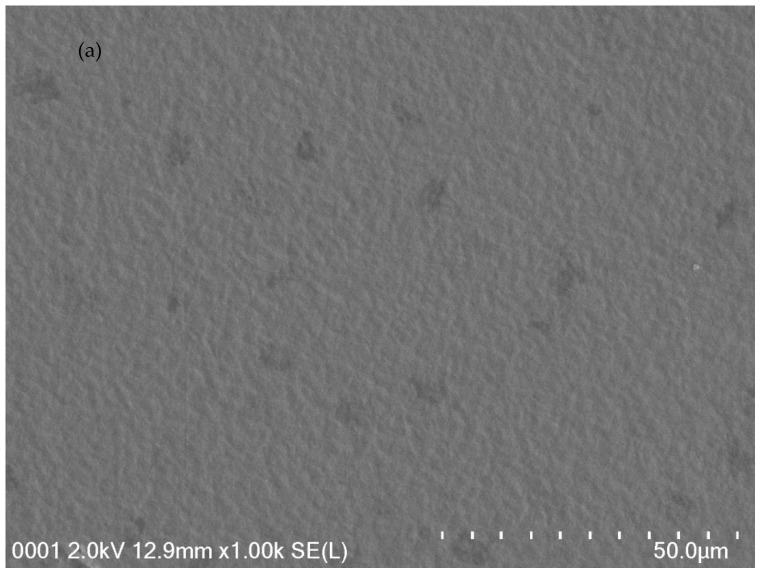

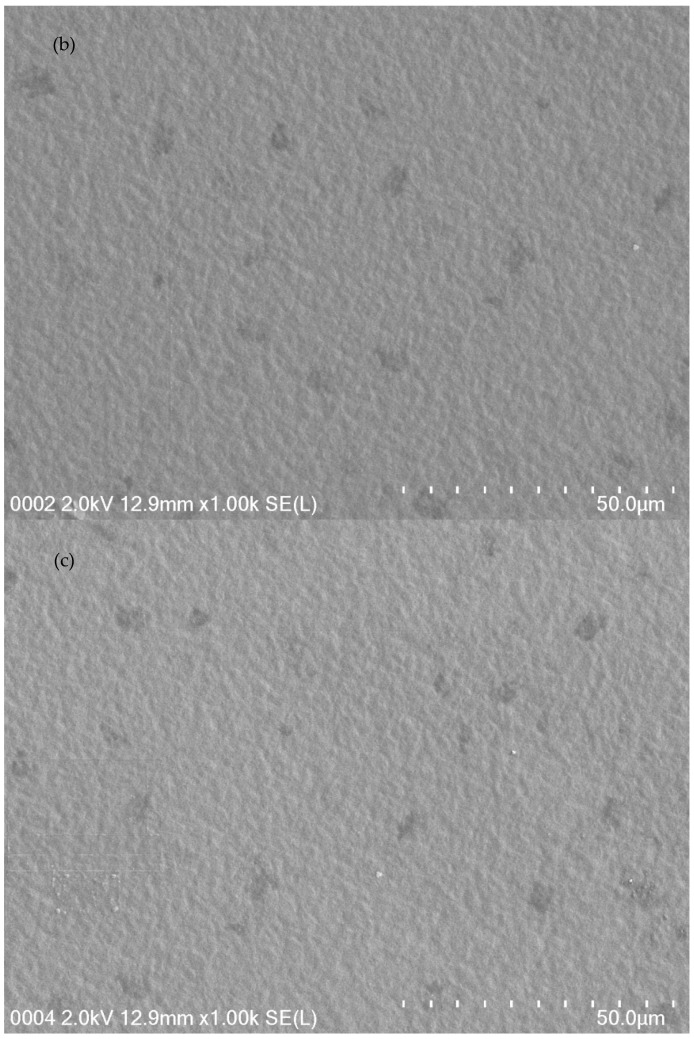

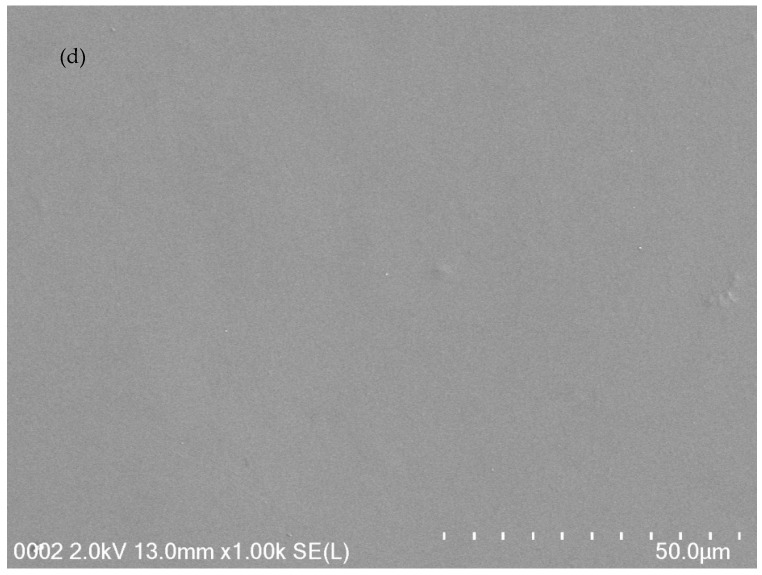
FESEM images for (**a**) CSDPB1, (**b**) CSDPB2, (**c**) CSDPB3, and (**d**) CSDPB4 blend electrolytes.

Dealing with material surface is vital to understand the structural changes over a number of processes. Recent studies revealed that the morphology aspect in polymers provides some insights into the changes in structural or electrical properties [9,25,28]. Polymer family can be categorized in terms of crystallinity into crystallineand amorphous polymers. On the one hand, polymer crystals are characterized by compact assembly of stereo-regular chains and thereby exhibit high modulus and hardness, but weak toughness. On the other hand, amorphous polymers features are rubbery or glassy in behavior [38]. In our previous work, SEM technique was used to study the compatibility of salts with polar polymers [39]. Figure 4a–d shows the FESEM images for a number of the CS:Dextran systems incorporated with various weight percentage of LiClO_4_ salt. The surface morphologies of the blend electrolyte samples are almost smooth and there are no protruded particles on the sample surfaces. This was observed in our previous work [40] where, as more salt is added, more particles protruded out of the surface. This indicates that polymer matrix capacity is limited to accommodate excess salt, which in turn led to salt recrystallization. It is apparent, as recrystallization proceeds, the amount of free ions lowers, which results in conductivity value decrement [41]. In fact, the smooth surface of the samples indicates that the complexation had taken place among the polymer blends and the incorporated LiClO_4_ salt. The data results of the present work indicate that polymer blend fabrication is a novel approach and straightforward methodology with high DC conductivity. The occurrence of the extent of complexation of the dopant salt and CS:Dextran was realized from the FTIR study.

### 2.3. Impedance Study

**Figure 5:**
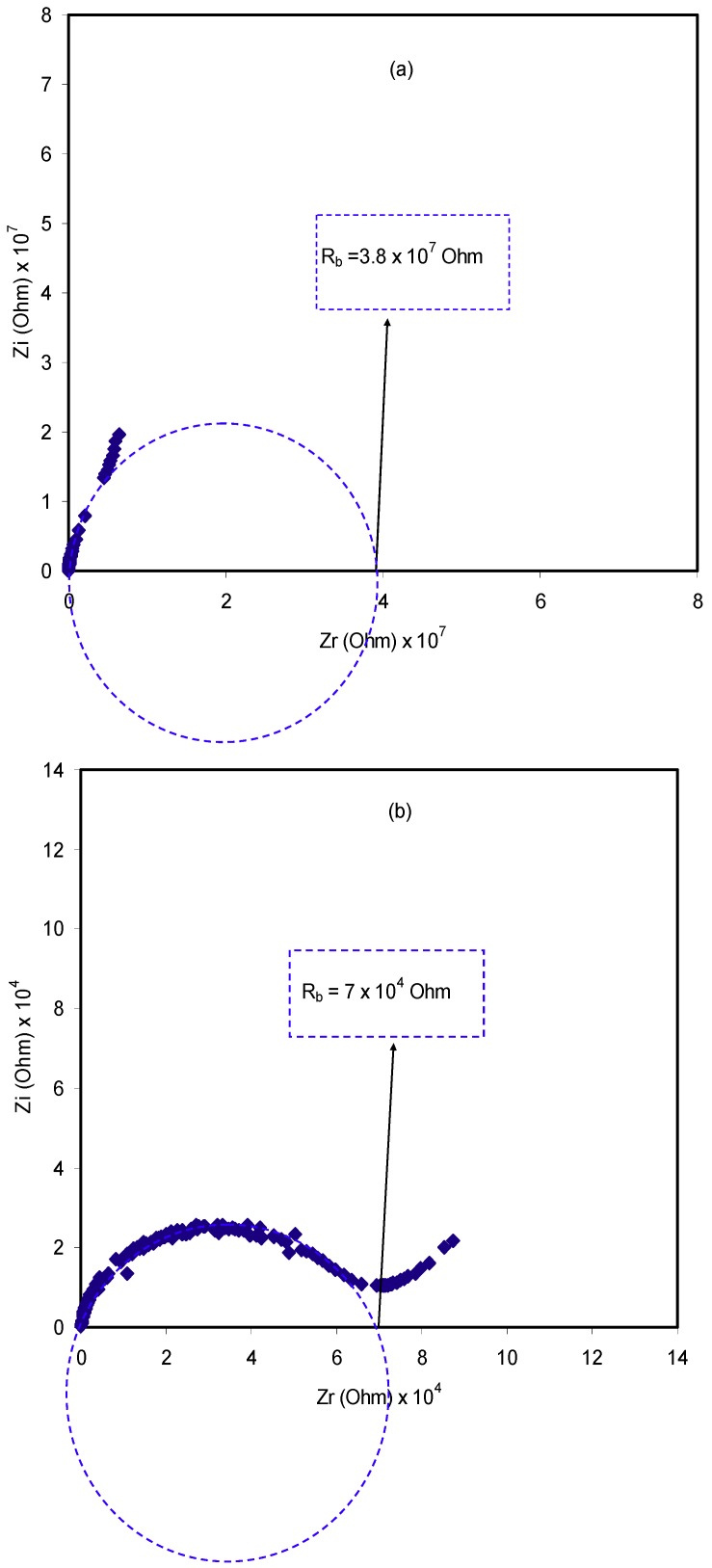

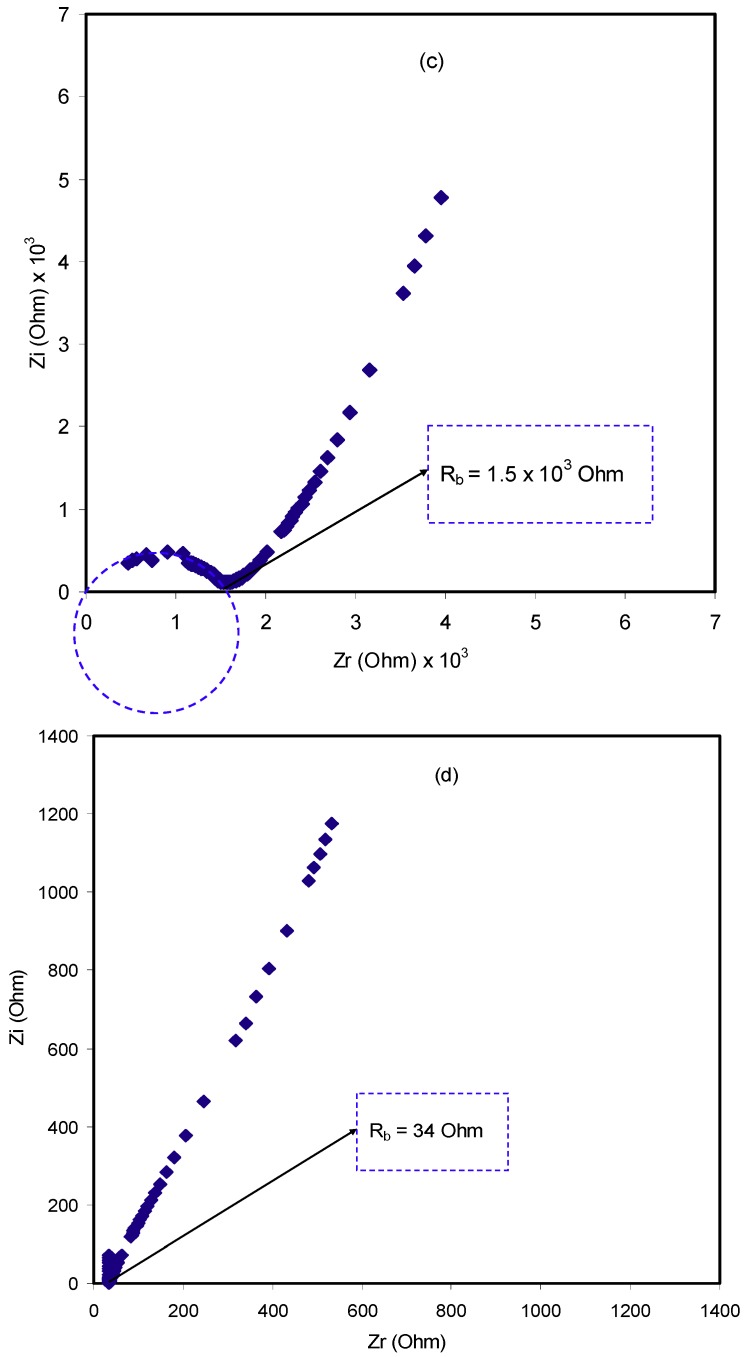

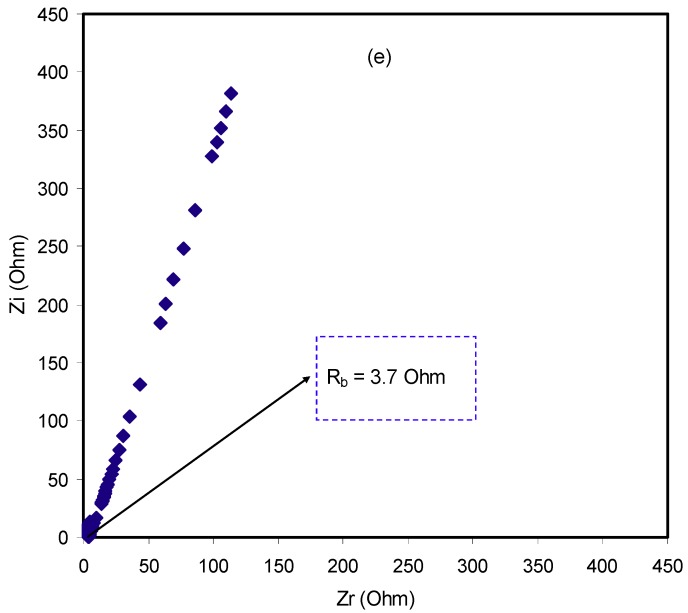
Experimental Impedance plots for (**a**) pure CS:Dextran film (**b**) CSDPB1, (**c**) CSDPB2, (**d**) CSDPB3, and (**e**) CSDPB4 blend electrolyte films.

A comparably new and powerful technique in the characterization of a number of the electrical properties of electrolyte materials and the interface region with electronically conducting electrodes is electrochemical impedance spectroscopy [28,42,43]. The impedance plot for CS:Dextran–LiClO_4_ polymer blend electrolyte systems at room temperature are shown in Figure 5a–d. The complex impedance plots reveal two main distinct regions: The semicircle observed at the high frequency region, which is due to the bulk character of the electrolytes, and the linear region at the low frequency range, which is attributed to the blocking electrodes [42,44,45]. The membrane electrolytes carry ion carriers, and thus, ion diffusion occurs through the membrane as an AC electric field is applied, and consequently, ion accumulation builds up at the electro/electrolyte interface. Due to the electronic nature of the stainless steel electrodes, ions cannot cross the system, and thus, the real and imaginary parts of the impedance can be measured at various frequencies, which resulted in impedance plots. It is interesting that, at the intermediate frequencies, certainly at −45 ~ inclined lines indicate the occurrence of Warburg impedance as a consequence of diffusion of ions to the electrode surface [46]. Furthermore, the spike feature at the low frequency region is a characteristic of diffusion process [29]. It is apparent, in Figure 5 that, with increasing salt concentration, the bulk resistance (see the insets) decreased. The *R_b_* value is determined by the point where the semicircle intersects the real axis (*Zr*). The equation below has been applied to determine the sample conductivity based on the *R_b_* value and the sample dimensions:

(1) $$\sigma_{dc}=\left(\frac{1}{R_{b}}\right)\times \left(\frac{t}{A}\right)$$

In the above equation, the polymer electrolyte film thickness and the film surface area are, respectively, denoted by *t* and *A*. Table 1 shows the calculated DC conductivity for all the samples. The high DC conductivity of blend electrolytes is a guarantee for EDLC application. To get more information about the charge transfer resistance of the samples Bode plots were also studied. More insights about the electrical properties of the blend electrolyte samples can be grasped from the modeling of the impedance plots using electrical equivalent circuits (EECs). Through the modeling of impedance plots, it is possible to estimate the bulk resistance and circuit elements. 

**Figure 6:**
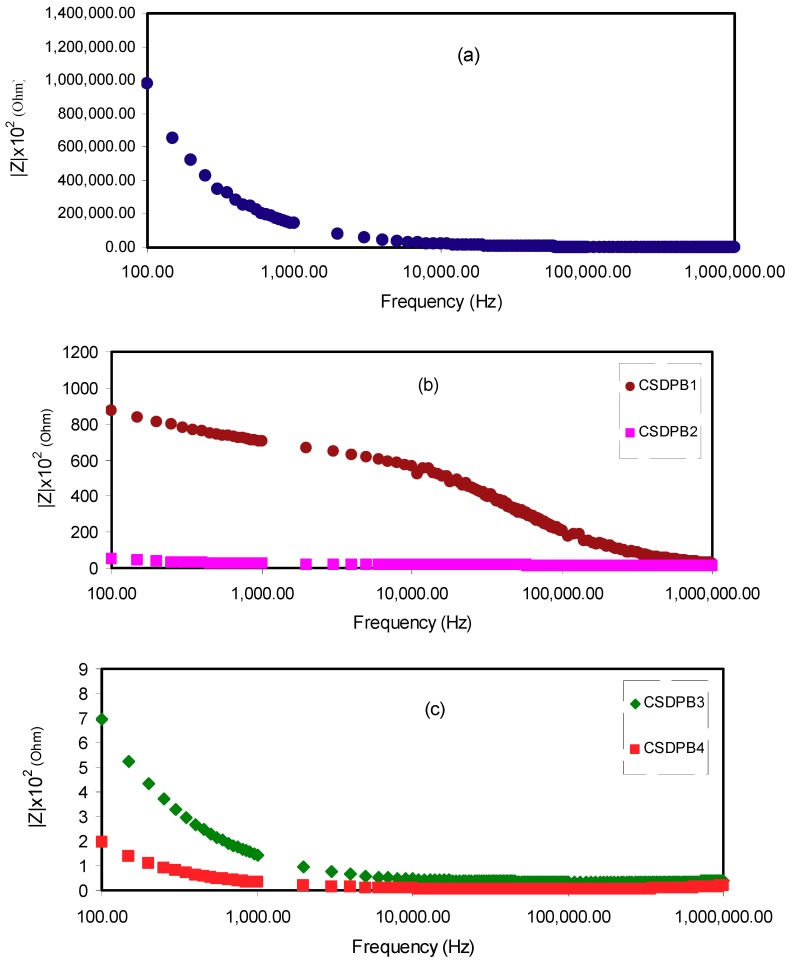
Bode plots for (**a**) pure CS:Dextran film (**b**) CSDPB1 and CSDPB2, and (**c**) CSDPB3, and CSDPB4 blend electrolyte films.

Moreover, the results of Nyquist plots are further established by the consideration of Bode plot. From the electrochemical viewpoint, Bode plots are principally helpful in understanding the charge transfer process in electrolyte materials [47]. Figure 6 shows the Bode plots for the pure CS:Dextran blend film and blend electrolyte films at ambient temperature. Previous studies have demonstrated that three distinguished regions should be recognized from the Bode plots, which are capacitive, diffusion, and charge transfer regions [39,47,48,49]. Usually, the capacitive region (namely plateau region) can be observed at a very low frequency; from 10^−2^ to 100 Hz. However, this region could not be examined in this study, owing to the frequency limitations of the measuring equipment. As described in the impedance plots of Figure 5, the semicircle has been correlated to the ion transfer in amorphous phase of electrolytes and the tails were related to the contribution of Warburg or diffusion of ions, and therefore, their accumulation at the electrode/electrolyte interface [40,43,45]. The ion accumulation on both sides of the electrolyte membrane will produce electrical double layer capacitances. The results clearly indicated that, with increasing salt concentration, from 10 to 40 wt.%, the Warburg contribution (tail regions) has been increased, and therefore, the resistance decreased due to the large amount of carrier density. It is obvious from Figure 6a that pure CS:Dextran films show high charge transfer resistance. Obviously, the charge transfer resistance decreases with increasing salt concentrations as shown in Figure 6b,c. From Figure 5 and Figure 6, it is clear that the sample incorporated with 40 wt.% of LiClO4 exhibits the lowest resistance and thus a high conductivity resulted.

**Figure 7:**
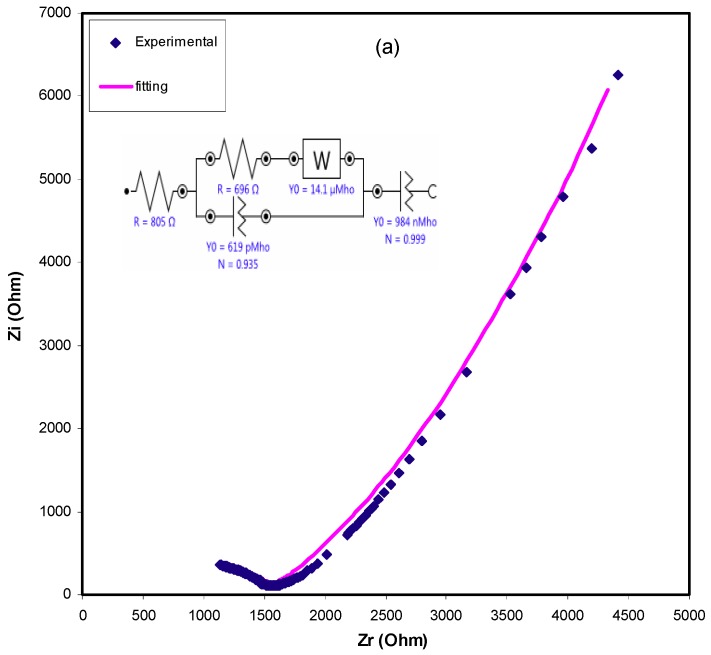

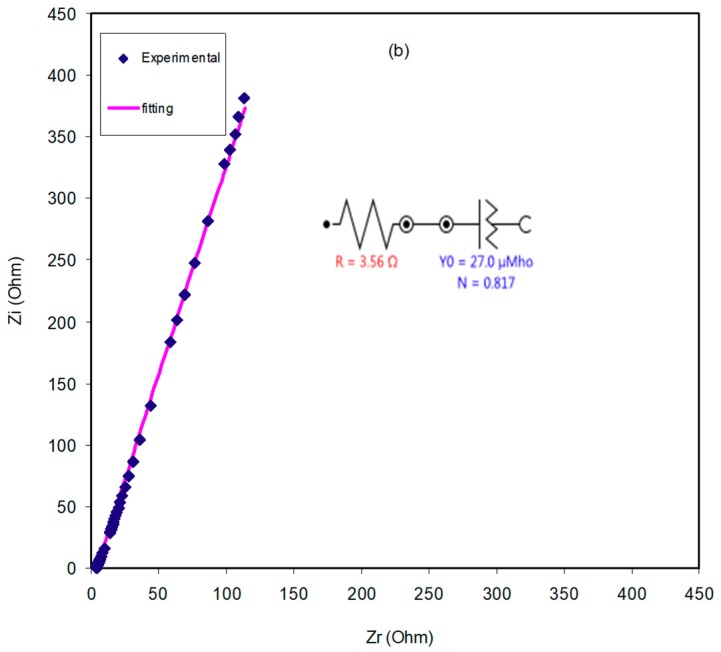
Experimental Impedance and fitting (EEC) plots for (**a**) CSDPB2 and (**b**) CSDPB4 blend electrolyte films.

The EECs model can be usually utilized for fitting, i.e., it can be used in the analysis of impedance spectroscopy, since the model is straightforward, quick, and provides a complete picture of the system [50]. The acquired impedance plots can be interpreted with respect to the equivalent circuit including *R_b_* for the charge carriers in the sample and two constant phase elements (CPE), as presented in the insets of Figure 7a. The high frequency region shows the combination of *R_b_* and CPE, whilst the low frequency region exhibits CPE, i.e., the developed double layer capacitance between the electrodes and SPE. The abbreviated term CPE is more commonly used in the equivalent circuit rather than as an ideal capacitor in real system. This is due to the fact that the behavior of the actual SPE is varied from that of an ideal capacitor considered in a pure semicircular pattern [51]. As discussed above, Warburg impedance at −45 ~ inclined lines are definitely a consequence of diffusion of ions to the electrode surface. In this report, the depressed semicircle has been explained by CPE instead of a capacitor [52]. The impedance of *Z_CPE_* can be written as [53,54]:

(2) $$Z_{CPE}=\frac{\cos(\pi n/2)}{Y_{m}\omega^{n}}-j\frac{\sin(\pi n/2)}{Y_{m}\omega^{n}}$$

where *Y_m_* refers to the CPE capacitance, *ω* is the angular frequency and *n* is associated to the deviation of the vertical axis of the plot in the complex impedance plots. Finally, the real (*Z_r_*) and imaginary (*Z_i_*) values of complex impedance (Z*) related with the equivalent circuit (insets of Figure 7a) can be expressed as:

(3) $$Z_{r}=R_{s}+\frac{R_{1}+R_{1}^{2}Y_{1}\omega^{n1}\cos(\pi n_{1}/2)}{1+2R_{1}Y_{1}\omega^{n_{1}}\cos(\pi n_{1}/2)+R_{1}^{2}Y_{1}^{2}\omega^{2n_{1}}}+\frac{\cos(\pi n_{2}/2)}{Y_{2}\omega^{n_{2}}}$$

(4) $$Z_{i}=\frac{R_{1}^{2}Y_{1}\omega^{n_{1}}\sin(\pi n_{1}/2)}{1+2R_{1}Y_{1}\omega^{n_{1}}\cos(\pi n_{1}/2)+R_{1}^{2}Y_{1}^{2}\omega^{2n_{1}}}+\frac{\sin(\pi n_{2}/2)}{Y_{2}\omega^{n_{2}}}$$

All circuit element parameters that are used for fitting the experimental impedance plots for all the selected samples are presented in Table 2. These elements are exactly related to the parameters in the above equations, such that *Y_o_* corresponds to *Y_m_*, *N* corresponds to *n*_1_ and *n*_2_ according to CPE1 and CPE2 elements and R corresponds to R_1_. In the Cole–Cole plot, the semicircle disappears at a certain high salt concentration of the salt (see Figure 7b), suggesting that only the resistive component of the polymer prevails [55]. In this case, the values of *Z_r_* and *Z_i_* associated to the EEC can be expressed as:

(5) $$Z_{r}=R+\frac{\cos(\pi n/2)}{Y_{m}\omega^{n}}$$

(6) $$Z_{i}=\frac{\sin(\pi n/2)}{Y_{m}\omega^{n}}$$

### 2.4. Transference Number Measurement (TNM) Study 

The ionic (*t_i_*) and electronic (*t_e_*) transference number can be gained from the ratio of steady-state current (*I_ss_*) and initial current (*I_i_*). The value of *t_i_* can be calculated from the following equation [55]:

(7) $$t_{i}=\frac{I_{i}-I_{ss}}{I_{i}}$$

**Figure 8:**
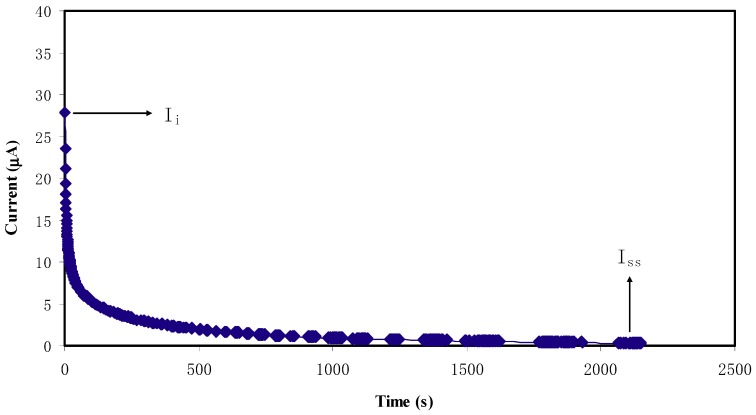
Polarization current versus time for the CSDPB4 blend electrolyte film.

Figure 8 shows the polarization plot of current versus time for the highest conducting electrolyte. The current at initial time is large due to migration of both ions and electrons. It is seen that the current is decreased rapidly prior to achieving a constant value of 0.4 μA, since only electron can pass through the stainless steel electrodes. At steady state, the cell is polarized as the current flow is stayed due to electron [56]. The constant current value indicates ionic conductor behavior of the electrolyte [57], where *I_ss_* and *I_i_* are observed to be at 0.4 and 27.8 μA, respectively. Therefore, *t_i_* and *t_e_* transference number for the electrolyte are found to be 0.98 and 0.02, respectively. This confirms the fact that ion is the dominant charge carrier in the electrolyte. Othman et al. [58] documented *t_i_* values from 0.93 to 0.98 for poly methyl methacrylate (PMMA)-lithium trifluoro methane sulfonate (LiCF_3_SO_3_). Therefore, the high transference number may be correlated with the effect of polymer-ion and ion-ion interactions on the microscopic parameter.

### 2.5. LSV Analysis

**Figure 9:**
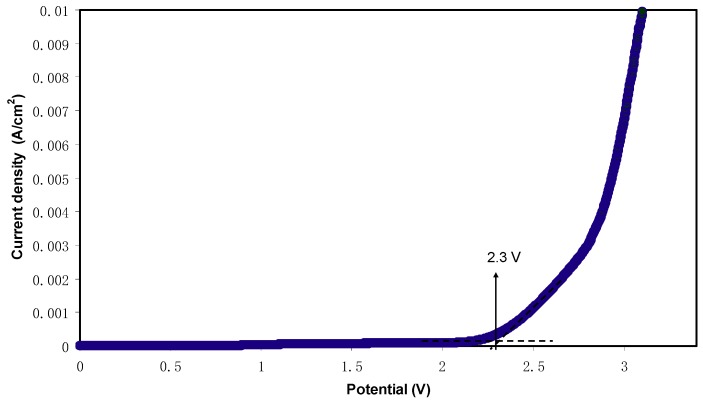
LSV plot for the highest conducting chitosan-dextran-LiClO_4_ (CSDPB4) sample.

Determination of potential stability is an imperative characteristic of the polymer electrolyte for energy storage device applications. Figure 9 shows the LSV plot of the highest conducting (CSDPB4) electrolyte. It is noticeable that there is no current flowing below 2.3 V, which indicates that there is no electrochemical reaction occurring below this potential window. The increase of current beyond 2.3 V is related to the decomposition of the polymer electrolyte, signifying the electrochemical reaction within the polymer electrolyte [59]. Monisha et al. [60] stated that the threshold voltage is that the current flows through the cells. Shukur et al. [52] reported a decomposition voltage at 2.10 V of lithium salt based biopolymer electrolyte. Thus, the potential stability, i.e., potential window of the relatively high conducting electrolyte in this work is suitable for energy storage device applications.

### 2.6. EDLC Study

#### CV and EDLC Characteristics

**Figure 10:**
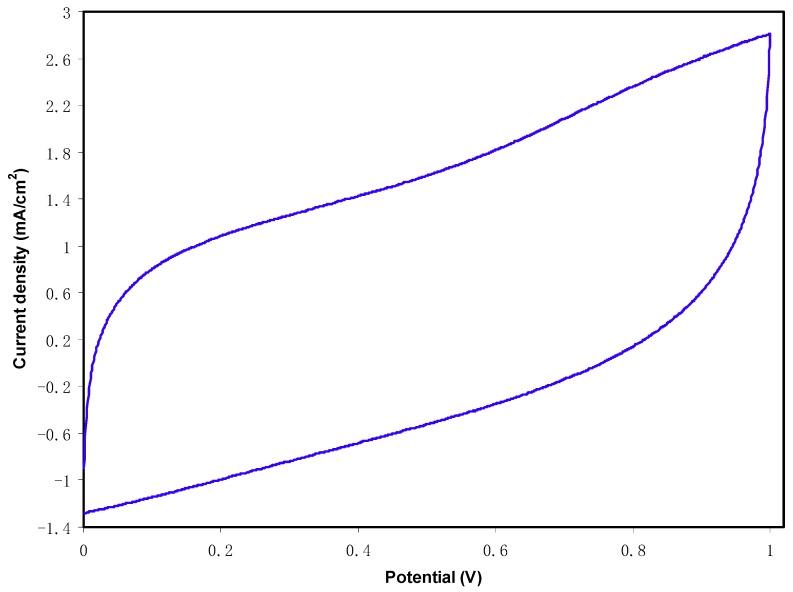
CV plot of the fabricated EDLC in the potential range of 0 V to 1 V.

Figure 10 shows the cyclic voltammogram of the fabricated EDLC at a sweep rate of 10 mV·s^−1^. It can be observed that there is no peak within the potential range of 0 to 1.0 V. However, there is an electrical double layer, i.e., non-Faradaic current at the surface of the electrodes [61]. As stated previously, energy storage mechanism in EDLC goes via non-Faradaic, which means that no redox reaction process. The addition of salt in the electrolyte produces positively charged ion (i.e., cations) and negatively charged ions (i.e., anions). Once the EDLC is charged, cations and anions will migrate to negative and positive electrodes, respectively. At negative electrode, the induced electric field at the electrode attracts cations and repels anions, where the opposite action takes place at the positive electrode. The intense electric field holds ions from the electrolyte and electrons from the electrode. This is called as development of charge double-layer, where it stores the energy as potential energy [62]. The shape of CV in Figure 10 depicts rapid switching of ions at the electrode electrolyte interfaces as well the good capacitive behavior of electrodes. The internal resistance and electrode porosity resulted in current dependence of voltage and makes the shape of the CV plot a less perfect rectangular [63]. 

**Figure 11:**
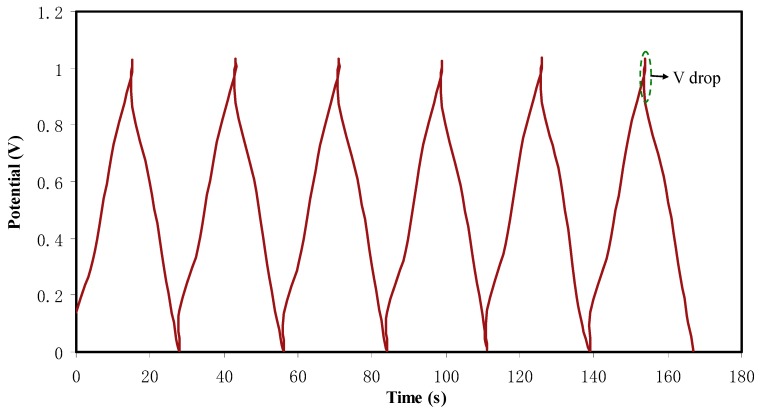
Charge-discharge profiles for the fabricated EDLC at 0.5 mA·cm^−2.^

**Figure 12:**
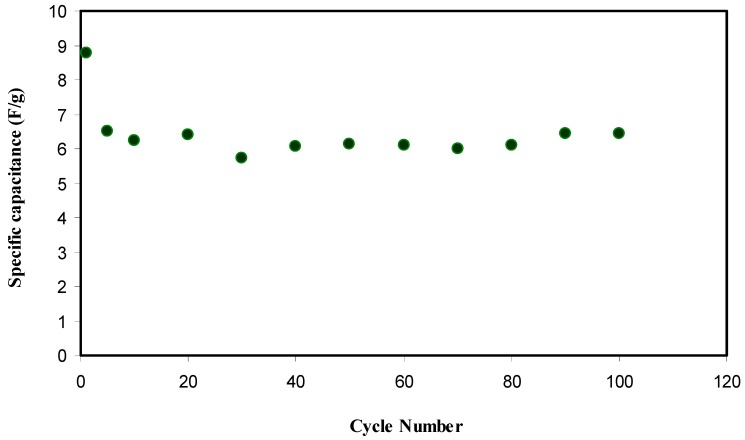
Specific capacitance of the fabricated EDLC for 100 cycles.

The charge–discharge profiles of the fabricated EDLC are investigated through galvanostatic technique. Figure 11 shows the charge-discharge plot of the fabricated EDLC at 0.5 mA·cm^−2^ in the potential range of 0 to 1 V. The discharge slope is observed to be almost linear, which verifies the capacitive behavior in the EDLC [64]. As the slope of the discharge curve (*s*) is determined, the specific capacitance (*C_s_*) can be calculated from the following equation:

(8) $$C_{S}=\frac{i}{sm}$$

here, *i* is the constant current and *m* stands for active material mass, which is the mass of active carbon in this study. In Figure 12, *C_s_* of the EDLC for 100 cycles can be seen, in which *C_s_* of the EDLC at the 1st cycle is found to be 8.7 F·g^−1^. Teoh et al. [65] have recorded a *C_s_* of 7.1 F·g^−1^ for free plasticizer LiClO_4_ based corn starch polymer electrolyte EDLC type capacitor. At 5th cycle, the *C_s_* drops to 6.5 F·g^−1^ and remains constant in the range of 6.0 to 6.5 F·g^−1^. This capacitance synthesized in the present work is of the great interest compared to the specific capacitance values of 2.6–3.0 and 1.7–2.1 F·g^−1^, which have been recorded for the EDLC cells with the Mg- and Li-based PEO polymer electrolytes incorporated with ionic liquids [66]. Therefore, polymer blend electrolytes can be established as new materials in fabricating EDLC cells with high specific capacitances.

**Figure 13:**
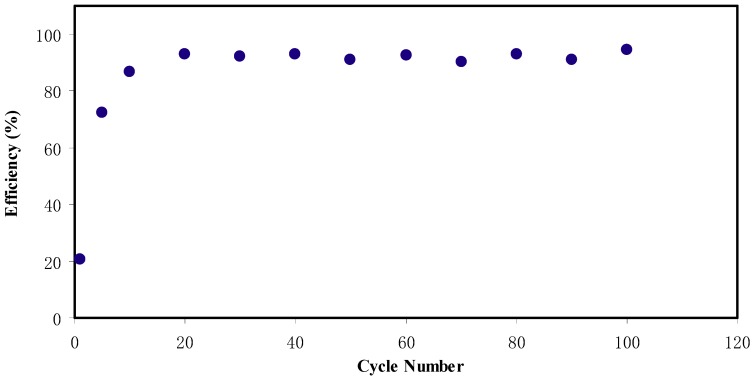
Cycling stability of the EDLC up to 100 cycles.

Coulombic efficiency (*η*) is another imperative parameter regarding the cycling stability of the EDLC where it can be calculated from the following equation:

(9) $$\eta =\frac{t_{d}}{t_{c}}\times 100$$

where discharge and charge time are denoted as *t_d_* and *t_c_*, respectively. Figure 13 shows the *η* of the EDLC of 100 cycles. The coulombic efficiency, *η* at the 1st cycle is found to be 20.6% and increases to 72.2% and 86.6% at 5th and 10th cycles, respectively. At 20th cycle, *η* is observed to be 92.8% and then lowered and remained constant at 92.0% up to 100 cycles. It is considered that the EDLC possesses plausible electrode-electrolyte contact as the *η* is beyond 90.0% [67].

As observed in Figure 11, there are tiny potential drops (*V_d_*) before the discharging process commences. This can be related to the existence of internal resistance in the EDLC, which is called equivalent series resistance (*R_esr_*). This resistance *R_esr_* of the EDLC can be obtained from the following equation:

(10) $$R_{esr}=\frac{V_{d}}{i_{}}$$

**Figure 14:**
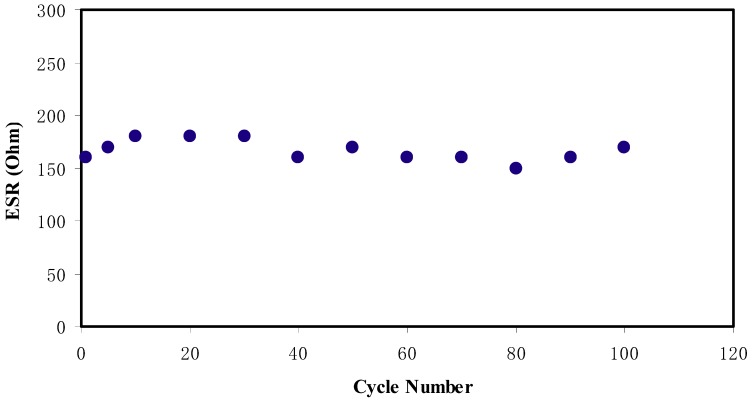
The pattern of equivalent series resistance of the EDLC for 100 cycles.

Figure 14 shows the *R_esr_* of the EDLC for 100 cycles. It is determined that the *R_esr_* varies from 150 to 180 Ω over the 100 cycles. The existence of internal resistance has been assumed to be due to various factors. The first one is from the electrolyte, where fast charge/discharge process cause free ions to recombine and reduce the ionic conductivity. Secondly, it is from current collectors, which in this case is the aluminum foil. Lastly, it is the gap between the electrolyte and electrode, where ions from the electrolyte and electrons from the carbon electrode form a charge double layer or called potential energy [68].

**Figure 15:**
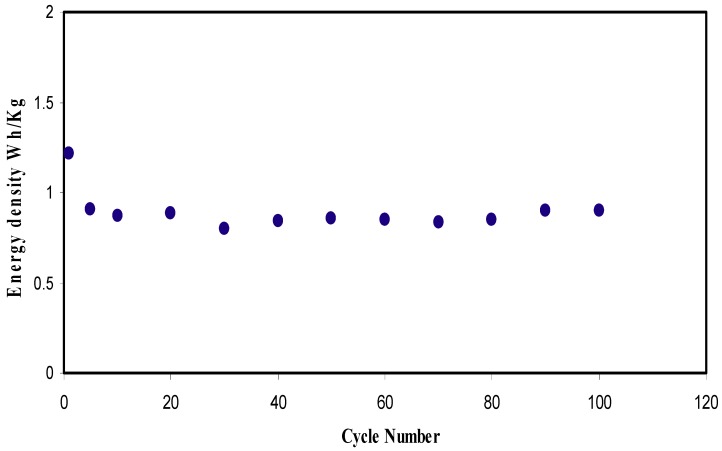
Energy density of the fabricated EDLC for 100 cycles.

**Figure 16:**
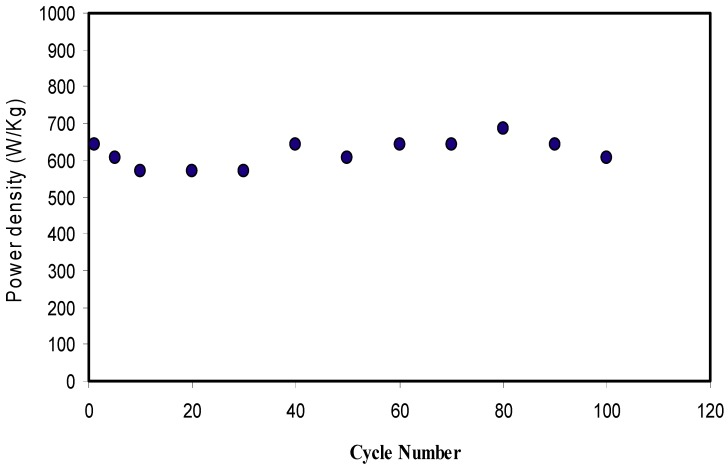
Power density of the fabricated EDLC for 100 cycles.

The energy density (*E_d_*) and power density (*P_d_*) of the EDLC can be expressed as:

(11) $$E_{d}=\frac{C_{s}V}{2}$$

(12) $$P_{d}=\frac{V^{2}}{4mR_{esr}}$$

where *V* is the applied voltage (1 V). In Figure 15, *E_d_* is found to be 1.21 Wh·kg^−1^ at the 1st cycle and then lowered to 0.90 Wh·kg^−1^ at 5th cycle. *E_d_* is then kept stable at 0.86 Wh·kg^−1^ up to 100 cycles. The *E_d_* values of the EDLC are almost constant, which harmonized with the pattern of *C_s_*. The stabilization study showed that the ions experience the same energy barrier during the migration towards the electrodes from 10th to 100th cycle of charge-discharge process [69]. The energy density achieved for the EDLC cell (0.86 Wh/Kg) in the present work is of great interest compared to that reported (0.3 Wh/Kg) for ionic liquid incorporated PEO based polymer electrolyte [66]. Figure 16 exhibits the *P_d_* of the EDLC for 100 cycles. The value of *P_d_* of the EDLC is found to be 643 W·kg^−1^ at the 1st cycle and dropped to 571 W·kg^−1^ at 10th cycle. The interesting observation is that the value of *P_d_* increases back to 685 W·kg^−1^ up to 80cycles. In Figure 14, *R_esr_* of the EDLC is also increased at 10th cycle and decreased until 80th cycle. At 90th and 100th cycles, the EDLC experiences reduction in *P_d_* value. This could be related to the growth of ion aggregates/pairs and electrolyte depletion during rapid charge-discharge process. The main drawbacks of polymer electrolyte membranes are ion aggregates/pairs. In this case little ions diffuse to the electrode/electrolyte interface. Consequently, the accumulated ion in the forming double-layer, at the surface of the electrodes, is reduced, and thus, reducing the power density [61].

## 3. Experimental Method

### 3.1. Materials and Sample Preparation

High molecular weight chitosan (CS) (average molecular weight 310,000–375,000) and Dextran powder (average molecular weight 35,000–45,000) materials were used as the raw materials (Sigma-Aldrich, Warrington, PA, USA). For the fabrication of the polymer blending based on CS:Dextran, 60 wt.% chitosan and 40 wt.% dextran was dissolved separately in 50 mL of 1% acetic acid at room temperature for 90 min. Subsequently, these solutions then mixed and stirred for 3 h to gain a homogeneous blending solution. For the blended solution of CS:Dextran, various amounts of LiClO_4_ ranging from 10 to 40 wt.% of LiClO_4_ in steps of 10 was added separately with continuous stirring to prepare CS:Dextran: LiTf electrolytes. The polymer blend electrolytes were coded as CSDPB1, CSDPB2, CSDPB3, and CSDPB4 for CS:Dextran and incorporated with 10, 20, 30, and 40 wt.% of LiClO_4_, respectively. After casting in different Petri dishes, the solutions were left to dry at room temperature for films to form. The films were transferred into a desiccator for further drying, which produces solvent-free films. The thickness of the SPBEM was from 0.0123 to 0.0131 cm.

### 3.2. TNM and LSV Measurements

**Figure 17:**
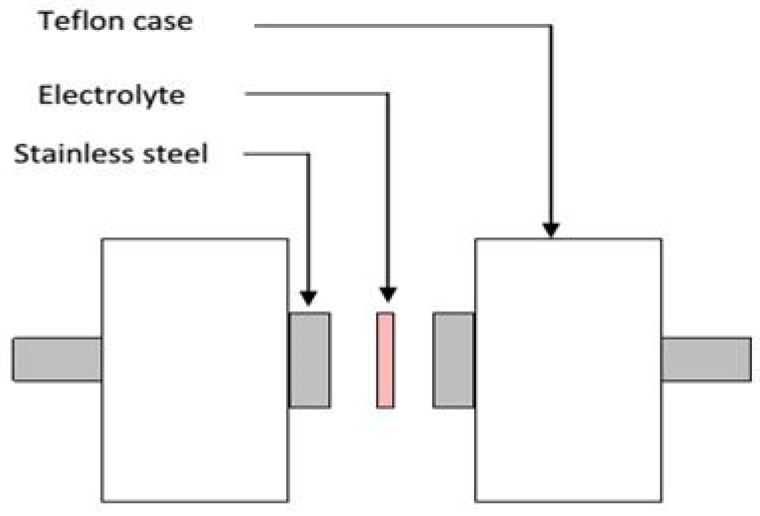
Schematic diagram of cell arrangement for LSV, TNM and impedance study.

V&A Instrument DP3003 digital DC power supply was employed to conduct the transference number (TNM) analysis via DC polarization method [70]. The cell was polarized at 30 mV and the DC current was monitored continuously as a function of time at room temperature. Stainless steel was used as electrodes for TNM due to its ion-blocking characteristic. Ionic and electronic transference numbers analyses are used to observe the contribution of ion and electron to the total conductivity and prove ionic conduction. The potential stability of the highest conducting electrolyte (CSDPB4) was studied using linear sweep voltammetry (LSV) (DY2300 Potentiostat) at sweep rate of 50 mV·s^−1^. For the LSV analysis, stainless steel was also used as counter, working and reference electrodes. For both LSV and TNM, the highest conducting electrolyte (CSDPB4) was placed in between two stainless steels in a Teflon conductivity holder, as shown in Figure 17.

### 3.3. EDLC Preparation

**Figure 18:**
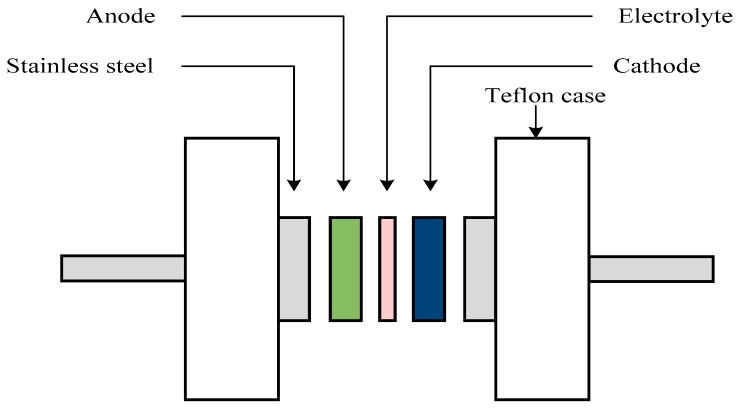
Diagram of the EDLC cell for CV and galvanostatic charge-discharge measurement.

An amount of 0.50 g polyvinylidene fluoride (PVdF) was dissolved with stirring in 15 mL *N*-methyl pyrrolidone (NMP). On the other hand, activated carbon (3.25 g) and carbon black (0.25 g) powders were dry mixed. The dry mixing process was carried out by using a planetary ball miller (XQM-0.4). Six metal balls were inserted in the chamber along with the powders. The powders were mixed at rotational speed of 500 r/min for 15 min. The powders were then added to the obtained solution of PVdF/NMP and stirred for 90 min. The homogeneous solution was doctor bladed on aluminum foil and heated at 60 °C for a period of time. The electrodes were then stored in desiccator filled with silica gel. The thickness of the electrode was 0.01 cm. The highest conducting electrolyte was sandwiched between two carbon electrodes, which were cut into circle shape with area of 2.01 cm^2^ and packed in CR2032 coin cells. The schematic diagram of the EDLC cell is shown in Figure 18. The galvanostatic charge-discharge characteristics of the EDLC were carried out using a battery cycler (Neware, Shenzhen, China) with a current density of 0.5 mA·cm^−2^. Digi-IVY DY2300 Potentiostat was used to conduct cyclic voltammetry (CV) of the EDLC at 10 mV·s^−1^ in the potential window of 0–1 V.

### 3.4. Structural, Morphological, and Impedance Characterizations

Spotlight 400 Perkin-Elmer spectrometer was used in conducting Fourier transform infrared (FTIR) spectroscopy with a resolution of 1 cm^−1^ (450–4000 cm^−1^). The surface of the electrolyte was analyzed via Hitachi SU8220 FESEM with 10K× magnification. For structural analysis, XRD pattern was acquired via D5000 X-ray diffractometer (1.5406 Å). The 2θ angle was continuously altered from 5° to 80° (resolution = 0.1°). In the mechanism study, HIOKI 3532–50 LCR HiTESTER was employed to analyze electrical impedance spectroscopy (EIS) measurements of the samples (50 Hz to 5 MHz). The cell arrangement for EIS is shown in Figure 17.

## 4. Conclusions

Solid polymer blend electrolytes (SPBE) composed of chitosan and dextran incorporated with various amounts of lithium perchlorate (LiClO_4_) was prepared. The relatively highest ion conducting sample was utilized to fabricate EDLC supercapacitor. Non-crystalline behavior of the polymer blend electrolytes has been confirmed from XRD pattern. The FTIR emphasized the strong interaction between the constituents of polymer electrolyte. The relatively smooth surface morphology of the electrolyte was found to be an indication of compatible LiClO_4_/polymer system. Faradaic process has shown to be absent and has definitely been noticed in the cyclic voltammetry. The conductivity of the samples was measured using impedance spectroscopy. The ionic (*t_i_*) and electronic (*t_e_*) transference number for the highest conducting electrolyte were found to be 0.98 and 0.02, respectively. The electrochemical stability window, as estimated from cyclic voltammetry, was found to be around 2.25 V for the Li^+^ ion conducting electrolyte. The absence of a peak in CV plot indicating the presence of electrical double layer at the surface of the electrodes. The highest ion conducting samples was used to fabricate the EDLC supercapacitor. The discharge slope has been observed to be almost linear, which verified that the EDLC possesses the capacitive behavior. The performance of fabricated EDLC was also studied using cyclic voltammetry and charge–discharge techniques. The highest specific capacitance was achieved to be at 8.7 F·g^−1^. At 20th cycle, the efficiency (*η*) was observed at 92.8%, where it remained constant at 92.0% up to 100 cycles. It was found that the EDLC possesses good electrode-electrolyte contact as *η* is above 90.0%. Another key finding is that the *R_esr_* was quite low and varied from 150 to 180 Ω over the 100 cycles. The energy density was quite high and equal to 1.21 Wh·kg^−1^ at the 1st cycle and then kept stable at 0.86 Wh·kg^−1^ up to 100 cycles. Lastly, the value of *P_d_* was also found to increase, returning to 685 W·kg^−1^ up to 80 cycles.

## Figures and Tables

**Table 1:** DC conductivity for pure CS:Dex and blend electrolyte films at room temperature.

Sample Designation	DC Conductivity (S/cm)
CS:Dex	5.01 × 10^−10^
CSDPB1	2.73 × 10^−7^
CSDPB2	1.27 × 10^−5^
CSDPB3	5.62 × 10^−4^
CSDPB4	5.16 × 10^−3^

**Table 2:** The parameters of the circuit elements of the selected blend electrolyte membranes at ambient temperature.

Sample	*R*_1_/*Ohm*	*Y*_1_	*n*_1_	*R_s_*	*Y*_2_/*nMho*	*n*_2_
CSDPB2	696	619 pMho	0.9345	805	984	0.99
CSDPB4	3.56	27 μMho	0.817	-	-	-

## References

[1] Iro Z.S., Subramani C., Dash S.S. (2016). A Brief Review on Electrode Materials for Supercapacitor. Int. J. Electrochem. Sci..

[2] Kiamahalleh M.V., Zein S.S.H., Najafpour G., Sata S.A., Buniran S. (2012). Multiwalled carbon nanotubes based nanocomposites for supercapacitors: A review of electrode materials. Nano.

[3] Shobana V., Parthiban P., Balakrishnan K. (2015). Lithium based battery-type cathode material for hybrid supercapacitor. J. Chem. Pharm. Res..

[4] Kamarudin K.H., Hassan M., Isa M.I.N. (2018). Lightweight and Flexible Solid-State EDLC based on Optimized CMC-NH4NO3 Solid Bio-Polymer Electrolyte. ASM Sci. J. Spec. Issue.

[5] Shukur A., Fadhlullah M. (2015). Characterization of Ion Conducting Solid Biopolymer Electrolytes Based on Starch-Chitosan Blend and Application in Electrochemical Devices. Ph.D. Dissertation.

[6] Kadir M.F.Z., Salleh N.S., Hamsan M.H., Aspanut Z., Majid N.A., Shukur M.F. (2017). Biopolymeric electrolyte based on glycerolized methyl cellulose with NH4Br as proton source and potential application in EDLC. Ionics.

[7] Aziz S.B., Hamsan M.H., Abdullah R.M., Kadir M.F.Z. (2019). A Promising Polymer Blend Electrolytes Based on Chitosan: Methyl Cellulose for EDLC Application with High Specific Capacitance and Energy Density. Molecules.

[8] Wang H., Lin J., Shen Z.X. (2016). Polyaniline (PANi) based electrode materials for energy storage and conversion. J. Sci. Adv. Mater. Devices.

[9] Hamsan M.H., Aziz B., Shukur M.F., Kadir M.F.Z. (2019). Protonic cell performance employing electrolytes based on plasticized methylcellulose-potato starch-NH4NO3. Ionics.

[10] Nyuk C.M., Isa M.I.N. (2018). Solid biopolymer electrolytes based on carboxymethyl cellulose for use in coin cell proton batteries. J. Sustain. Sci. Manag..

[11] Salleh N.S., Aziz S.B., Aspanut Z., Kadir M.F.Z. (2016). Electrical impedance and conduction mechanism analysis of biopolymer electrolytes based on methyl cellulose doped with ammonium iodide. Ionics.

[12] Hassan M.F., Azimi N.S.N., Kamarudin K.H., Sheng C.K. (2018). Solid Polymer Electrolytes Based on Starch-Magnesium Sulphate: Study on Morphology and Electrical Conductivity. ASM Sci. J. Spec. Issue.

[13] Du B.W., Hu S.Y., Singh R., Tsai T.T., Lin C.C., Ko F.U. (2017). Eco-Friendly and Biodegradable Biopolymer Chitosan/Y_2_O_3_ Composite Materials in Flexible Organic Thin-Film Transistors. Materials.

[14] Moniha V., Alagar M., Selvasekarapandian S., Sundaresan B., Hemalatha R., Boopathi G. (2018). Synthesis and characterization of bio-polymer electrolyte based on iota-carrageenan with ammonium thiocyanate and its applications. J. Solid State Electrochem..

[15] Netsopa S., Niamsanit S., Sakloetsakun D., Milintawisamai N. (2018). Characterization and Rheological Behavior of Dextran from Weissella confusa R003. Int. J. Polym. Sci..

[16] Sarwat F., Ahmed N., Aman A., Qader S.A.U. (2013). Optimization of growth conditions for the isolation of dextran producing Leuconostoc spp. from indigenous food sources Pak. J. Pharm. Sci..

[17] Barsbay M., Guner A. (2007). Miscibility of dextran and poly(ethylene glycol) in solid state: Effect of the solvent choice, Carbohydr. Polymers.

[18] Telegeev G., Kutsevo N., Chumachenko V., Naumenko A., Telegeeva P., Filipchenko S., Harahuts Y. (2017). Dextran-Polyacrylamide as Matrices for Creation of Anticancer Nanocomposite. Int. J. Polym. Sci..

[19] Misenan M.S.M., Isa M.I.N., Khiar A.S.A. (2018). Electrical and structural studies of polymer electrolyte based on chitosan/methyl cellulose blend doped with BMIMTFSI. Mater. Res. Express.

[20] Hamsan M.H., Shukur M.F., Kadir M.F.Z. (2017). The effect of NH4NO3 towards the conductivity enhancement and electrical behavior in methyl cellulose-starch blend based ionic conductors. Ionics.

[21] Kharbachi A.E., Hu Y., Yoshida K., Vajeeston P., Kim S., Sørby M.H., Orimo S., Fjellvåg H., Hauback B.C. (2018). Lithium ionic conduction in composites of Li(BH4)0.75I0.25 and amorphous 0.75Li2S0.25P2S5 for battery applications. Electrochim. Acta.

[22] Tamilselvi P., Hema M. (2014). Conductivity studies of LiCF3SO3 doped PVA: PVdF blend polymer electrolyte. Physica B.

[23] Yusof Y.M., Shukur M.F., Illias H.A., Kadir M.F.Z. (2014). Conductivity and electrical properties of corn starch–chitosan blend biopolymer electrolyte incorporated with ammonium iodide. Phys. Scr..

[24] Kadir M.F.Z., Hamsan M.H. (2018). Green electrolytes based on dextran-chitosan blend and the effect of NH4SCN as proton provider on the electrical response studies. Ionics.

[25] Aziz S.B., Abidin Z.H.Z., Kadir M.F.Z. (2015). Innovative method to avoid the reduction of silver ions to silver nanoparticles in silver ion conducting based polymer electrolytes. Phys. Scr..

[26] Aziz S.B., Kadir M.F.Z., Abidin Z.H.Z. (2016). Structural, morphological and electrochemical impedance study of CS:LiTf based solid polymer electrolyte: Reformulated Arrhenius equation for ion transport study. Int. J. Electrochem. Sci..

[27] Hamsan M.H., Shukur M.F., Aziz S.B., Kadir M.F.Z. (2019). Dextran from Leuconostoc mesenteroides-doped ammonium salt-based green polymer electrolyte. Bull. Mater. Sci..

[28] Aziz S.B., Abidin Z.H.Z., Arof A.K. (2010). Effect of silver nanoparticles on the DC conductivity in chitosan–silver triflate polymer electrolyte. Physica B.

[29] Malathi J., Kumaravadivel M., Brahmanandhan G.M., Hema M., Baskaran R., Selvasekarapandian S. (2010). Structural, thermal and electrical properties of PVA–LiCF3SO3 polymer electrolyte. J. Non-Cryst. Solids.

[30] Aziz S.B. (2016). Role of dielectric constant on ion transport: Reformulated Arrhenius equation. Adv. Mater. Sci. Eng..

[31] Shukla R., Shukla S., Bivolarski V., Iliev I., Ivanova I., Goyal A. (2011). Structural Characterization of Insoluble Dextran Produced by Leuconostoc mesenteroides NRRL B-1149 in the Presence of Maltose. Food Technol. Biotechnol..

[32] Vettori M.H.P.B., Franchetti S.M.M., Contiero J. (2012). Structural characterization of a new dextran with a low degree of branching produced by Leuconostoc mesenteroides FT045B dextransucrase. Carbohydr. Polym..

[33] Dumitraşcu M., Meltzer V., Sima E., Vîrgolici M., Albu M.G., Ficai A., Moise V., Minea R., Vancea C., Scărişoreanu A. (2011). Characterization of electron beam irradiated collagenpolyvinylpyrrolidone (PVP) and collagen-dextran (DEX) blends. Dig. J. Nanomater. Biostruct..

[34] Aziz S.B., Abidin Z.H.Z. (2013). Electrical conduction mechanism in solid polymer electrolytes: New concepts to arrhenius equation. J. Soft Matter.

[35] Nikoli G.S., Caki M., Miti Z., Ili B., Premovic P. (2009). Attenuated Total Reflectance–Fourier Transform Infrared Microspectroscopy of Copper(II) Complexes with Reduced Dextran Derivatives. Russian J. Phys. Chem. A.

[36] Wei D., Sun W., Qian W., Ye Y., Ma X. (2009). The synthesis of chitosan-based silver nanoparticles and their antibacterial activity. Carbohydr. Res..

[37] Mitić Ž., Cakić M., Nikolić G. (2010). Fourier-Transform IR spectroscopic investigations of Cobalt(II)–dextran complexes by using D2O isotopic exchange. Spectroscopy.

[38] Han C.C., Shi W., Jin J. (2013). Morphology and Crystallization of Crystalline/Amorphous Polymer Blends. Encyclopedia Polym. Compos..

[39] Aziz S.B., Abdullah R.M., Kadir M.F.Z., Ahmed H.M. (2019). Non suitability of silver ion conducting polymer electrolytes based on chitosan mediated by barium titanate (BaTiO_3_) for electrochemical device applications. Electrochim. Acta.

[40] Aziz S.B., Abdullah O.G., Rasheed M.A., Ahmed H.M. (2017). Effect of high salt concentration (HSC) on structural, morphological, and electrical characteristics of chitosan based solid polymer electrolytes. Polymers.

[41] Shukur M.F., Kadir M.F.Z. (2015). Hydrogen ion conducting starch-chitosan blend based electrolyte for application in electrochemical devices. Electrochim. Acta.

[42] Polu A.R., Kumar R. (2011). AC impedance and dielectric spectroscopic studies of Mg2+ionconducting PVA–PEG blended polymer electrolytes. Bull. Mater. Sci..

[43] Aziz S.B., Woo T.J., Kadir M.F.Z., Ahmed H.M. (2018). A conceptual review on polymer electrolytes and ion transport models. J. Sci. Adv. Mater. Devices.

[44] Aziz S.B., Abidin Z.H.Z., Arof A.K. (2010). Influence of silver ion reduction on electrical modulus parameters of solid polymer electrolyte based on chitosan-silver triflate electrolyte membrane. ExpressPolym. Lett..

[45] Aziz S.B. (2018). The mixed contribution of ionic and electronic carriers to conductivity in chitosan based solid electrolytes mediated by CuNt Salt. J. Inorg. Organomet. Polym..

[46] Hirankumar G., Selvasekarapandian S., Bhuvaneswari M.S., Baskaran R., Vijayakumar M. (2004). AC Impedance Studies on Proton Conducting Polymer Electrolyte Complexes (PVA+CH3COONH 4). Ionics.

[47] Aziz S.B., Faraj M.G., Abdullah O.G. (2018). Impedance Spectroscopy as a Novel Approach to Probe the Phase Transition and Microstructures Existing in CS:PEO Based Blend Electrolytes. Sci. Rep..

[48] Eftekhari A. (2018). The mechanism of ultrafast supercapacitors. J. Mater. Chem. A..

[49] Eftekhari A. (2019). Surface Diffusion and Adsorption in Supercapacitors. ACS Sustain. Chem. Eng..

[50] Pradhan D.K., Choudhary P., Samantaray B.K., Karan N.K., Katiyar R.S. (2007). Effect of Plasticizer on Structural and Electrical Properties of Polymer Nanocompsoite Electrolytes. Int. J. Electrochem. Sci..

[51] Mohapatra S.R., Thakur A.K., Choudhary R.N.P. (2009). Effect of nanoscopic confinement on improvement in ion conduction and stability properties of an intercalated polymer nanocomposite electrolyte for energy storage applications. J. Power Sources.

[52] Shukur M.F., Ithnin R., Kadir M.F.Z. (2014). Electrical characterization of corn starch-LiOAc electrolytes and application in electrochemical double layer capacitor. Electrochim. Acta.

[53] Aziz S., Abdullah R.M. (2018). Crystalline and amorphous phase identification from the tanδ relaxation peaks and impedance plots in polymer blend electrolytes based on [CS: AgNt]x:PEO (x−1)(10 ≤ x ≤ 50). Electrochim. Acta.

[54] Teo L.P., Buraidah M.H., Nor A.F.M., Majid S.R. (2012). Conductivity and dielectric studies of Li2SnO3. Ionics (Kiel).

[55] Hema M., Selvasekarapandian S., Arunkumar D., Sakunthala A., Nithya H.F.T.I.R. (2009). FTIR, XRD and ac impedance spectroscopic study on PVA based polymer electrolyte doped with NH4X (X = Cl, Br, I). J. Non-Cryst. Solids.

[56] Kufian M.Z., Aziz M.F., Shukur M.F., Rahim A.S., Ariffin N.E., Shuhaimi N.E.A., Arof A.K. (2012). PMMA-LiBOB gel electrolyte for application in lithium ion batteries. Solid State Ionics.

[57] Diederichsen K.M., McShane E.J., McCloskey B.D. (2017). McCloskey Promising Routes to a High Li+ Transference Number Electrolyte for Lithium Ion Batteries. ACS Energy Lett..

[58] Othman L., Isa K.B., Osman Z., Yahya R. (2013). Ionic Conductivity, Morphology and Transport Number of Lithium Ions in PMMA Based Gel Polymer Electrolytes. Defect Diffus. Forum.

[59] Sampathkumar L., Selvin P.C., Selvasekarapandian S., Perumal P., Chitra R., Muthukrishnan M. (2019). Synthesis and characterization of biopolymer electrolyte based on tamarind seed polysaccharide, lithium perchlorate and ethylene carbonate for electrochemical applications. Ionics.

[60] Monisha S., Mathavan T., Selvasekarapandian S., Benial A.M.F., Premalatha M. (2017). Preparation and characterization of cellulose acetate and lithium nitrate for advanced electrochemical devices. Ionics.

[61] Liew C.W., Ramesh S. (2015). Electrical, structural, thermal and electrochemical properties of corn starch-based biopolymer electrolytes. Carbohydr. Polym..

[62] Kadir M.F.Z., Arof A.K. (2013). Application of PVA–chitosan blend polymer electrolyte membrane in electrical double layer capacitor. Mater. Res. Innov..

[63] Bandaranayake C.M., Weerasinghe W.A.D., Vidanapathirana K.P., Perera K.S. (2015). A Cyclic Voltammetry study of a gel polymer electrolyte based redox-capacitor. Sri. Lankan J. Phys..

[64] Teoh K.H., Liew C.W., Ramesh S. (2014). Electric double layer capacitor based on activated carbon electrode and biodegradable composite polymer electrolyte. Ionics.

[65] Teoh K.H., Lim C.S., Liew C.W., Ramesh S. (2015). Electric double-layer capacitors with corn starch-based biopolymer electrolytes incorporating silica as filler. Ionics.

[66] Pandey G.P., Kumar Y., Hashmi S.A. (2011). Ionic liquid incorporated PEO based polymer electrolyte for electrical double layer capacitors: A comparative study with lithium and magnesium systems. Solid State Ionics.

[67] Lim C.S., Teoh K.H., Liew C.W., Ramesh S. (2014). Capacitive behavior studies on electrical double layer capacitor using poly (vinyl alcohol)-lithium perchlorate based polymer electrolyte incorporated with TiO_2_. Mater. Chem. Phys..

[68] Arof A.K., Kufian M.Z., Syukur M.F., Aziz M.F., Abdelrahman A.E., Majid S.R. (2012). Electrical double layer capacitor using poly(methyl methacrylate)–C4BO8Li gel polymer electrolyte and carbonaceous material from shells of mata kucing (Dimocarpus longan) fruit. Electrochim. Acta.

[69] Hamsan M.H., Shukur M.F., Kadir M.F.Z. (2017). NH_4_NO_3_ as charge carrier contributor in glycerolized potato starch-methyl cellulose blend-based polymer electrolyte and the application in electrochemical double-layer capacitor. Ionics.

[70] Shukur M.F., Ithnin R., Kadir M.F.Z. (2014). Protonic transport analysis of starch-chitosan blend based electrolytes and application in electrochemical device. Mol. Cryst. Liq. Cryst..

